# Genes and Diseases: Insights from Transcriptomics Studies

**DOI:** 10.3390/genes13071168

**Published:** 2022-06-28

**Authors:** Dmitry S. Kolobkov, Darya A. Sviridova, Serikbai K. Abilev, Artem N. Kuzovlev, Lyubov E. Salnikova

**Affiliations:** 1 The Laboratory of Ecological Genetics, Vavilov Institute of General Genetics, Russian Academy of Sciences, Moscow 119991, Russia; dmitry.s.kolobkov@gmail.com (D.S.K.); daria_sv11@mail.ru (D.A.S.); abilev@vigg.ru (S.K.A.); 2 The Laboratory of Clinical Pathophysiology of Critical Conditions, Federal Research and Clinical Center of Intensive Care Medicine and Rehabilitology, Moscow 107031, Russia; artem_kuzovlev@fnkcrr.ru; 3 The Laboratory of Molecular Immunology, Rogachev National Research Center of Pediatric Hematology, Oncology and Immunology, Moscow 117997, Russia

**Keywords:** differential expression (DE), disease genes, gene expression omnibus (GEO), gene ontology (GO), transcriptomics analysis of healthy tissues, human phenotype ontology (HPO)

## Abstract

Results of expression studies can be useful to clarify the genotype-phenotype relationship. However, according to data from recent literature, there is a large group of genes that are revealed as differentially expressed (DE) in many studies, regardless of the biological context. Additional analyses could shed more light on the relationships between genes, their differential expression, and diseases. We generated a set of 9972 disease genes from five gene-phenotype databases (OMIM, ORPHANET, DDG2P, DisGeNet and MalaCards) and a report of the International Union of Immunological Societies. To study transcriptomics of disease and non-disease genes in healthy tissues, we obtained data from the Human Protein Atlas (HPA) website. We analyzed the dependency between expression in healthy tissues and gene occurrence in Gene Expression Omnibus series using tools within the Enrichr libraries. The results of expression studies were annotated with Gene Ontology (GO) and Human Phenotype Ontology (HPO) terms. Using transcriptomics analysis of healthy tissues, we validated the previous findings of higher expression levels of disease genes in pathologically linked tissues compared to other tissues. Preferentially DE genes were generally highly expressed in one or multiple tissues and were enriched for disease genes. According to the results of GO enrichment analyses, both down- and up-regulated DE genes most often took part in immune response, translation and tissue-specific processes. A connection between DE-related pathology and the diversity of HPO terms was found. Investigating a link between expression and phenotype contributes to understanding the mode of development and progression of human diseases.

## 1. Introduction

Large-scale studies of gene expression are widely used to explore molecular mechanisms of human diseases. Most of these studies aim to identify genes with differential expression (DE) between diseased and healthy groups. It is generally accepted that an integrated analysis of DE genes can provide valuable information to identify disease markers and describe a targeted disease landscape in general [1,2,3]. However, recent studies demonstrated predictability of DE for a large group of genes, independent of the biological conditions in which they were investigated [4,5]. An analysis of overlapping genes within a framework of more than 600 expression studies showed that preferentially variable genes can be attributed to several sets of DE genes implicated in a common biological function, which were called DE modules. These modules included genes relating to sex, extracellular matrix, immune system and inflammation, cell cycle, and response to stress [4]. Since this finding is of a general nature, a number of questions arise. Could it be that main DE modules do not differ for different tissues and directions of effect (down- or up-regulation)? Is there a link between gene expression in normal tissues and DE in pathology affected tissues? Are there differences in DE predictability between genes associated with a disease and other genes? Does the number of DE findings of a gene in transcriptome-wide studies correlate with the number of phenotypic terms for a gene within and between phenotypic categories?

Some of these issues have been partially addressed in the literature. Disease-associated genes tend to be expressed at higher levels in those healthy tissues where alterations of these genes cause pathology [6]. Gene expression profiles across healthy organs correlated with organ-specific cancer incidence, in particular, a positive correlation with cancer incidence was demonstrated for highly expressed genes involved in transcription, translation, and protein synthesis [7]. Both down and up-regulated DE genes may be enriched in the same biological processes [8,9], possibly due to compensatory mechanisms [10,11]. In multifactorial disorders and drug-induced perturbations, characteristics of DE profiles correlated with the broadness of phenotypic manifestations [12].

Due to swift development of molecular genetic technologies and rapid accumulation of gene expression data in public repositories, progressive refinement of expression studies becomes possible. In this work, we sought to use modern resources to address relationships between gene expression in healthy tissues and DE in pathology studies. With this objective, we generated a large set of disease genes from five gene-phenotype databases (OMIM, ORPHANET, DDG2P, DisGeNet and MalaCards) and the International Union of Immunological Societies (IUIS) report [13]. Due to the role of the immune system in the maintenance of tissue homeostasis and organism integrity [14], special attention was paid to the genes associated with immune diseases. In subsequent analyses, immune disease genes were compared with genes associated with respiratory and nervous system diseases. We analyzed transcriptomic profiles of disease and non-disease genes in healthy tissues and blood cells and assessed their expression levels and tissue specificity. We investigated the dependency between expression in healthy tissues and gene occurrence as DE in the Gene Expression Omnibus (GEO) series within the Enrichr libraries Disease Perturbations from GEO down studies and Disease Perturbations from GEO up studies. Structured Gene Ontology (GO) and Human Phenotype Ontology (HPO) vocabularies were used to incorporate the data of expression studies in the context with translational impact.

## 2. Materials and Methods

### 2.1. Creating a Series of Disease Genes

OMIM, ORPHANET, DDG2P, DisGeNet, and MalaCards were used to generate a list of disease genes. OMIM phenotypes related to a particular gene were obtained from the Gene Map retrieval set https://www.omim.org/search/advanced/geneMap (accessed on 1 August 2021). and annotated with Clinical Synopsis data (https://www.omim.org/search/advanced/clinicalSynopsis) with the MIMmatch service (accessed on 20 August 2021). ORPHANET gene-disease pairs were downloaded from http://www.orphadata.org/cgi-bin/index.php (accessed on 1 August 2021). Rare disease classifications were available from http://www.orphadata.org/cgi-bin/rare_free.html (accessed on 8 August 2021). DDG2P gene diseases and attributes were retrieved from https://www.ebi.ac.uk/gene2phenotype/downloads (accessed on 8 June 2021). DisGeNet curated gene-disease associations (GDA) with GDA scores ≥ 0.3 were obtained from https://www.disgenet.org/downloads. The list of GDAs was further restricted to the evidence index (EI) ≥ 0.9 MalaCards https://www.malacards.org/ (accessed on 15 June 2021). elite genes with their corresponding diseases were kindly provided by MalaCards team (the MalaCards dump for GeneCards Suite Version v5.2). Elite genes are defined by gene-disease scores ≥ 25, computed as a weighted sum of individual scores derived from eight sources of information: OMIM, ClinVar, Orphanet, SwissProt-Humsavar, GeneTests, DISEASES, Novoseek, and GeneCards. The list of primary immunodeficiency (PID) genes was taken from the IUIS report [13]. A gene was considered to be related to a specific system disease (immune, respiratory or nervous) according to the classification in any of the resources used.

### 2.2. Transcriptomic Data in Healthy Tissuesand Tissue- and Blood Cell-Specific Gene Enrichment Analysis

We used data for tissue expression from the site ‘Downloadable data’ from Human Protein Atlas (HPA) homepage (https://www.proteinatlas.org/about/download (accessed on 20 August 2021)). The following data sets were downloaded and analyzed: HPA (43 tissues, *n* = 43) and GTEx (*n* = 34) sets for tissue gene data, and HPA (*n* = 19) and Monaco (*n* = 30) sets for blood cell gene data. All data are based on The Human Protein Atlas version 21.0 and Ensembl version 103.38. HPA sets included data on 19,670 genes, while GTEx and Monaco [15] sets included data on 18,816 genes. All GTEx and Monaco genes were also present in the HPA series. The TissueEnrich R package [16] was used to carry out tissue- and blood cell-specific gene enrichment analysis. Tissue enriched, group enriched, and tissue enhanced genes identified with TissueEnrich were grouped together under the name ‘tissue-specific genes’. The teEnrichment function (with default parameters) was applied to calculate the enrichment of tissue-specific genes in the groups of immune, respiratory, and nervous system disease genes [16].

### 2.3. Gene Occurrence in Pathology Transcriptomics from ENRICHR Libraries for GEO Studies

In this section, we determined how often each gene was detected as DE in studies samples for a specific tissue. Enrichr libraries Disease Perturbations from GEO down studies and Disease Perturbations from GEO up studies were downloaded from https://maayanlab.cloud/Enrichr/#libraries (accessed on 25 August 2021). After exclusion of non-human samples, there were 568 samples collected by crowding [17] from 339 studies. Gene names were checked with HGNC Multi-symbol Checker (http://www.genenames.org/cgi-bin/symbol_checker (accessed on 26 August 2021)) and revised so that one HGNC term combined all synonyms and corresponded to one stable ENSG ID (Ensembl Gene Identifier) (Ensembl version 106). Since stable ENSG ID are kept the same throughout Ensembl releases, ENSG IDs were further used to match genes from HPA and GEO studies. Tissues on which the studies were conducted were checked manually using the original descriptions of the studies on the GEO website. Furthermore, tissues were grouped by types to access the number of tissue-specific DE findings.

### 2.4. GO Enrichment Analysis

Gene Ontology enrichment analysis was used to functionally characterize DE genes. Enriched biological processes were considered in the sets of DE genes in blood/bone marrow, lung, cerebral cortex, skeletal muscle, and skin. Each sample was analyzed separately with two tools, Enrichr: GO Biological Process 2021 (https://maayanlab.cloud/Enrichr/ (accessed on 25 August 2021)) and STRING: GO Biological Process (https://string-db.org/ (accessed on 18 September 2021)). For gene set enrichment analyses, the false discovery rate (FDR) threshold was set at 0.05. We collected data on all enriched GO terms in all samples within tissue type and calculated the frequency of the occurrence of each GO term for the tissue. The REVIGO tool was used for data visualization and reducing redundancy of GO terms [18].

### 2.5. Mapping HPO Annotations to Genes

To link the results of transcriptomics studies with the variability of phenotypic abnormalities encountered in human genetic diseases, we used standardized terms for phenotypic abnormalities in hereditary diseases, which were obtained from Human Phenotype Ontology (HPO) [19]. The list of HPO gene-phenotype associations in all HPO diseases was downloaded from (https://ci.monarchinitiative.org/view/hpo/job/hpo.annotations/lastSuccessfulBuild/artifact/rare-diseases/util/annotation/genes_to_phenotype.txt (accessed on 19 July 2021)). Each HPO phenotype was mapped to a HPO category available through search and downloads of phenotypic terms relating to individual diseases. Categorization of the term did not depend on a specific disease. From the total of 27 categories in HPO, the categories “Inheritance” and “Clinical modifier” were excluded.

### 2.6. Identification of Haploinsufficient Genes

Among genes associated with genetic diseases, there are a relatively large fraction of haploinsufficient genes, which encode proteins required in large or highly titrated amounts. Haploinsufficiency can be defined as prediction of sensitivity to decreased gene dosage. The comparative analysis of haploinsufficient and haplosufficient genes could further clarify the connection between gene expression and variability of phenotypic manifestations of a disease. Haploinsufficiency was analyzed with DECIPHER, which provides haploinsufficiency scores calculated based on predicted haploinsufficiency probabilities (https://www.deciphergenomics.org/about/downloads/data (accessed on 18 October 2021)). Scores in the range 0–10% correspond to the highest probability of a gene to be haploinsufficient.

### 2.7. Statistical Analysis

To analyze differences between continuous variables in compared data sets, we used the non-parametric Mann–Whitney U test. It is used to test the null hypothesis that two compared samples were pooled from the same sample. FDR-corrected *p* values < 0.05 were considered significant.

## 3. Results

### 3.1. Disease Genes

**Figure 1:**
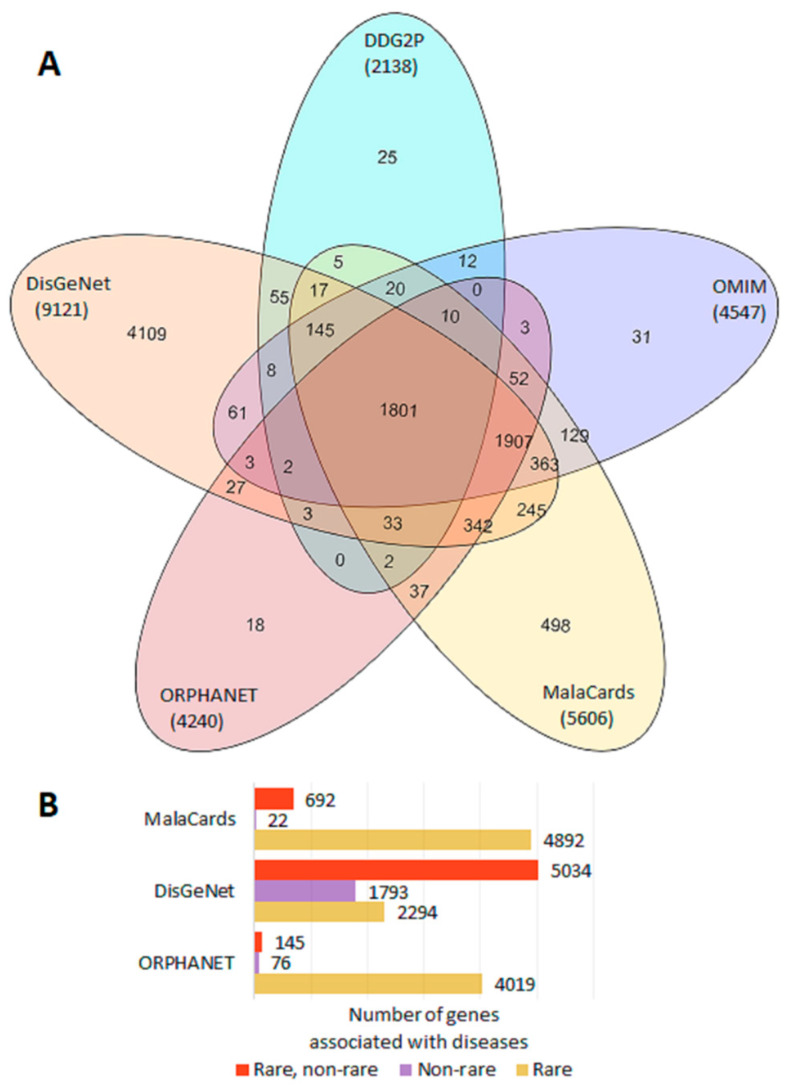
Representation of disease genes in gene–phenotype databases. (**A**) Venn diagram of gene sharing by OMIM, ORPHANET, DDG2P, DisGeNet, and MalaCards. (**B**) Number of genes associated with rare and non-rare diseases in ORPHANET, DisGeNet, and MalaCards.

From a total of 9972 genes with reliable proof of being associated with a disease, 9963 genes were extracted from OMIM, ORPHANET, DDG2P, DisGeNet and/or MalaCards (Figure 1A), and nine more genes were found in the IUIS report (Supplementary Tables S1 and S2). Classifications which allowed the identification of genes associated with rare and/or non-rare diseases were available in ORPHANET, DisGeNet, and/or MalaCards. The largest number of genes associated with non-rare diseases was presented in DisGeNet (Figure 1B, Supplementary Table S1).

### 3.2. Transcriptomic Profile of Disease- and Non-Disease Genes across Healthy Human Tissues and Blood Cells

**Figure 2:**
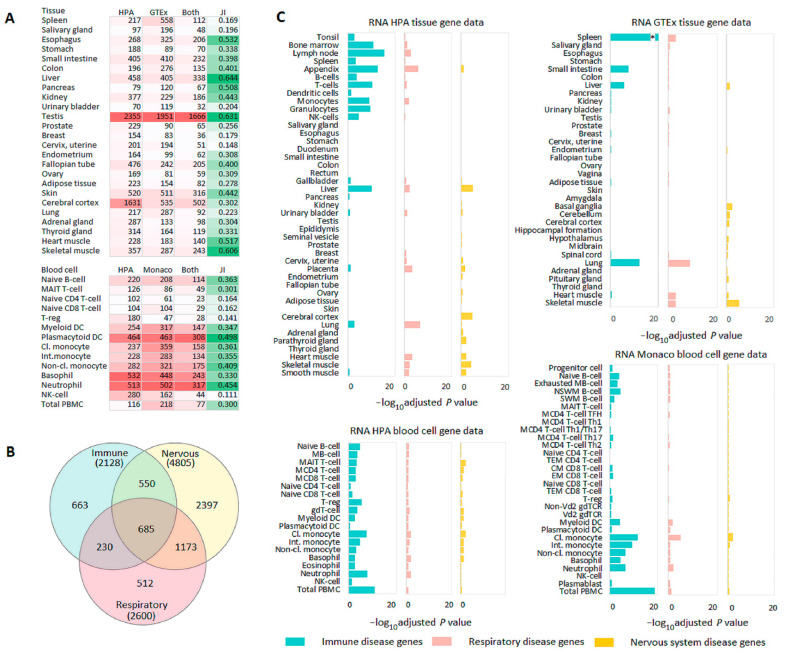
Tissue- and blood cell-specific gene enrichment analysis. (**A**) Heatmap with the number of genes (colored in red) enriched in the same tissues (HPA and GTEx) or blood cells (HPA and Monaco). Enriched genes include genes relating to all categories of enrichment in a corresponding tissue (tissue enriched, group enriched, and tissue enhanced). Jaccard index (JI) (colored in green) shows tissue or blood cell similarity determined by gene enrichment analysis with TissueEnrich. (**B**) Venn diagram showing an overlap among immune, respiratory, and nervous system disease genes. (**C**) TissueEnrich results for immune, respiratory, and nervous system disease genes. Abbreviations: Cl., Classical (monocyte); CM, Central memory (CD8 T-cell); EM, Effector memory (CD8 T-cell); Int., Intermediate (monocyte); M, Memory (MB-cell, MCD4 T-cell, MCD8 T-cell); Non-cl., Non-classical (monocyte); NSWM, Non-switched memory (B-cell); SWM, Switched memory (B-cell); TEM, Terminal effector memory (CD4, CD8 cells); * −log_10_ adjusted *p* value = 43.22.

We examined the transcriptomic profile in healthy tissues with the use of two tissue (HPA and GTEx) and two blood cell (HPA and Monaco) data sets. Correlation analysis revealed high Spearman correlation coefficients for all gene expression levels in the same tissues (*n* = 25, rho 0.89–0.95) and in the same blood cells (*n* = 14, rho 0.84–0.92). Identical tissues and blood cells are listed in Figure 2A. Expression profiles in TPM (Transcripts Per Million) were analyzed in specific system disease genes, other disease genes and non-disease genes (Supplementary Figures S1–S3, Supplementary Table S3). A comparison of expression levels of genes involved in the development of immune system diseases and genes associated with non-immune diseases showed that the former had higher levels of RNA expression in all immune related tissues and some other tissues (38/77 in two tissue sets) (Supplementary Table S3). Similar results were found in blood cells (43/49 in two blood cell sets). Lower expression levels were detected in four tissues (nervous and muscle tissues) whereas no differences were registered in 40 tissues, mainly urogenital, endocrine, and nervous (Supplementary Figure S1). Respiratory disease genes compared to non-respiratory disease genes had higher expression levels in eight tissues (8/77) with maximal differences detected in lung tissue (Supplementary Figure S2). Nervous system disease genes were expressed at higher levels than non-nervous system disease genes in 15 tissues (15/77) including nervous tissues (Supplementary Figure S3). In blood cell series, respiratory, and nervous system disease genes mainly did not differ by the level of expression from other disease genes. In all four tissue/blood cell sets, higher expression levels were typically observed for disease genes compared to non-disease genes (Supplementary Figures S1–S3).

Since gene-phenotype databases differ by sources for data collection and disease classifications, the consistency of transcriptomics results was assessed with a sensitivity analysis for two tissue (HPA and GTEx) data sets. The sequential exclusion of each of the five gene-phenotype databases did not have an essential impact on the results. Compared to other disease genes and non-disease genes, immune disease genes had higher expression levels in immune tissues, respiratory disease genes were expressed at higher levels in lungs, and nervous system disease genes were expressed at higher levels in nervous tissues. Some exceptions were mainly found for certain nervous system related tissues from the GTEx data set (Supplementary Table S4).

### 3.3. Tissue- and Blood Cell-Specific Gene Enrichment

TissueEnrich was used for tissue- and blood cell-specific gene enrichment analysis. First, we assessed the numbers of tissue-enriched genes and tissue similarity measured by the Jaccard index. The numbers of genes enriched in the same tissues (HPA and GTEx) or blood cells (HPA and Monaco) and Jaccard indices strongly differed (Figure 2A). The Jaccard index varied in the range of 0.15–0.63 for tissues and 0.11–0.50 for blood cells, which showed the diversity of transcriptomics sets. Next, we focused on tissue- and blood cell-specific gene enrichment analysis of immune, respiratory, and nervous system disease genes. Despite a rather strong overlap between these genes (Figure 2B), the gene set enrichment analysis showed characteristic enrichment of immune, respiratory, and nervous system disease genes in corresponding tissues from HPA and GTEx (Figure 2C). Immune disease genes were mainly enriched in immune related tissues (grouped together from tonsil to NK cells [20]), liver and lung; respiratory disease genes were primarily enriched in lung and also in several immune and muscle tissues; and nervous system disease genes were enriched in brain and muscle tissues. Immune disease genes were overrepresented in almost all blood cells in HPA and Monaco sets with the most significant results obtained for PBMCs. Compared to immune disease genes, respiratory and nervous system disease genes were poorly enriched in blood cells (Figure 2C). Thus, despite the diversity of transcriptomics sets, the results of the gene set enrichment analysis were concordant within both tissue- and blood cell transcriptomes. These results, in particular, supported the literature data on higher expression levels of disease genes in pathologically linked tissues compared to other tissues.

### 3.4. Gene Occurrence in DE Studies and Their Expression Profiles in Normal Tissues

GEO disease perturbations samples from the Enrichr libraries included data on 18,920 and 18,457 down- and up-regulated genes, respectively. Taking into account strong gene expression correlation among closely related tissues [21], we grouped all tissues, on which GEO studies were conducted by tissue types (Supplementary Table S5). After that, we selected the disease genes (Supplementary Table S1) in all GEO samples (9021/9972, 90.5% and approximately half of all genes included in the Enrichr libraries) (Supplementary Table S6) and demonstrated that they had a much larger total number of GEO associations and gene-unique tissue associations compared to non-disease genes (*p* value < 1 × 10^−300^) (Supplementary Figure S4). Then, we calculated the number of disease genes associated with phenotypes presented in GEO studies (*n* = 4848) and the number of gene–phenotype associations supported by expression changes (*n* = 955) (Supplementary Table S7). Out of 955 genes, 100 genes were both down and up-regulated. The percentage of samples with the associations (from the total number of samples per phenotype taking into account the analyzed tissue) was 24.4% and 22.4% for down- and up-regulated genes, respectively. Thus, the disease genes were actively involved in a variety of pathological processes, often unrelated to the diseases, to which they predispose.

**Figure 3:**
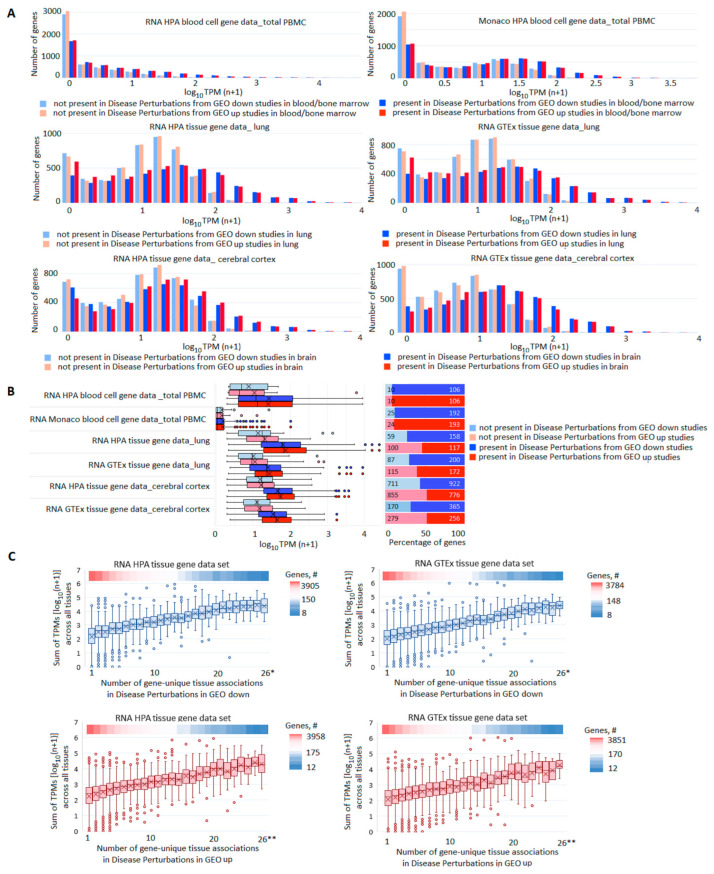
Comparison of gene expression profiles in norm and pathology. (**A**) Distribution of genes by their presence/absence in GEO studies’ libraries in dependence on their expression level in corresponding normal tissues. For all pairs of compared sets, the differences in expression levels between genes present or absent in GEO libraries were highly significant (*p* value < 1 × 10^−300^). (**B**) Expression levels (left panel) and the number (right panel) of tissue-specific genes present/absent among DE genes in GEO libraries. (**C**) Box plots reflecting correlations between the sum of TPMs across all tissues in HPA or GTEx data sets and the number of GEO tissues in which the expression of the gene changed. The upper heatmap band on each panel reflects the number of genes at each point; * 26–28; ** 26–30.

Next, we analyzed the pools of DE genes by assessing the expression of each gene in normal tissues and the number of gene records in the libraries of GEO studies. Figure 3A shows the distribution of genes by their presence/absence among DE genes in GEO samples on the relevant tissues (blood/bone marrow, lung, brain) as a function of their expression level in normal tissues (total PBMC from HPA and Monaco blood cells, lung, and cerebral cortex from HPA and GTEx tissues). The higher the gene expression was in a tissue (in normal state), the higher the probability was that it registered as changing expression between cases and controls. A similar phenomenon was recorded for tissue-specific genes (Figure 3B). Summarized TPMs for each gene across all tissues in HPA and GTEx were correlated with the number of gene-unique tissue associations in GEO samples (Figure 3C, Supplementary Table S6). Although tissue sets differed greatly, there was a moderate correlation (rho 0.39–0.44) between the sum of TPMs and the number of tissues, in which the expression of the gene changed.

### 3.5. GO Enrichment Analysis

**Figure 4:**
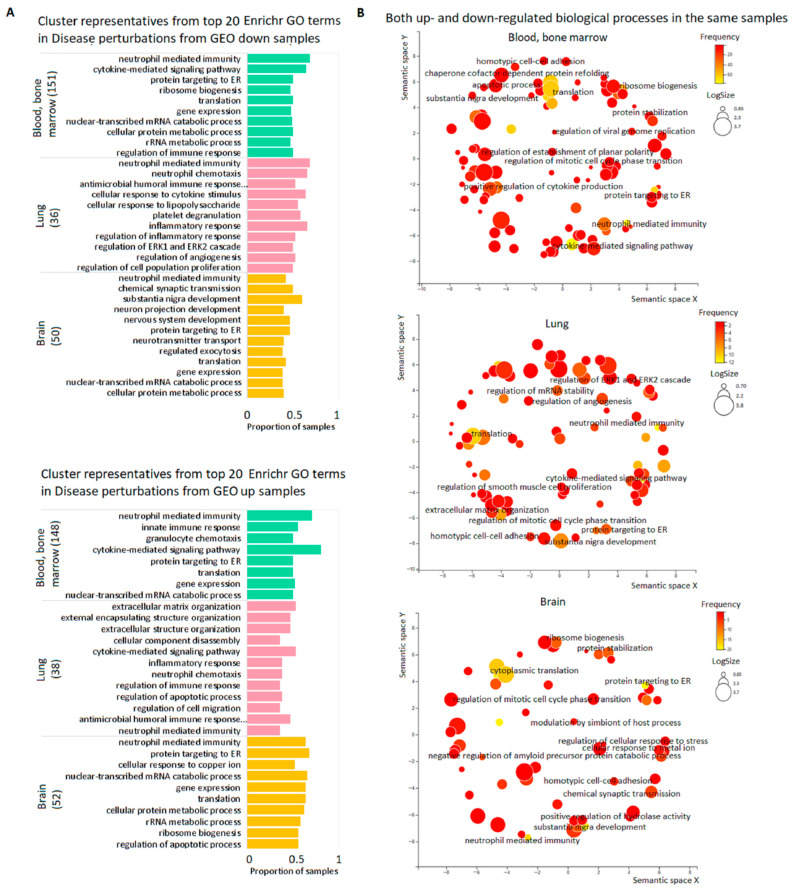
Top biological processes dysregulated in the pools of tissue samples. (**A**) Cluster representatives for the most frequent BP dysregulated in blood/bone marrow, lung, and brain. Cluster representatives for GO terms (terms remaining after reducing redundancy) were obtained using the REVIGO tool. The number of samples for each tissue is indicated in brackets. (**B**) REVIGO scatterplots for the same BP down-and up-regulated in the same samples. GO terms are plotted according to log size on the *x*-axis and frequency of occurrence (yellow is larger, red is smaller) in the samples on the *y*-axis. The size of the circles is proportional to the underlying frequency of the GO term in a reference database—the EBI GOA database. Functionally similar GO terms are located close to each other.

To uncover biological processes (BP) which are characteristic for DE genes in GEO studies performed on the selected tissues, we considered each sample separately with the use of GO tools. Some BP were typical for many samples in the same or even different tissues. Top 20 frequent terms in Enrichr GO Biological Process 2021 are presented in Figure 4A (blood/bone marrow, lung, and brain) and Supplementary Figure S5A (skeletal muscle and skin). The most frequent term for the genes down-regulated in blood/bone marrow, lung, and brain, namely ‘neutrophil-mediated immunity’, was represented in 68.2%, 68.4% and 42.31% of samples, respectively; for up-regulated genes, this term occurred in 70.9%, 36.1% and 64.0% of samples. Noteworthy, down- and up-regulated genes were frequently enriched in the same BP (Figure 4B, Supplementary Figure S5B). The results from STRING GO enrichment supported these observations (Supplementary Tables S8 and S9). The concordant results from the two GO enrichment tools showed that immune response, translation, and tissue-specific processes were most often disturbed in all considered tissues. The Enrichr results for the subsets of neoplastic diseases (Supplementary Table S5) fit these conclusions (Supplementary Table S10).

### 3.6. Gene-Based Analysis of Associated HPO Terms and Expression in Norm and Pathology

**Figure 5:**
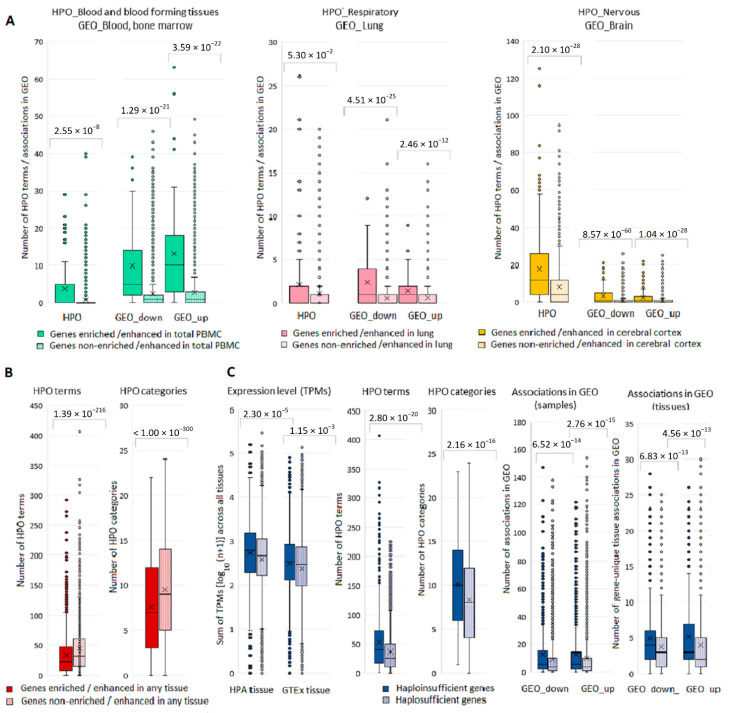
Box-plots depicting differences in the number of phenotypic associations and associations gene expression—disease in GEO for tissue specific and haploinsufficient genes. (**A**) Tissue-specific and non-specific genes with the number of terms within relevant phenotypic categories and the number of GEO samples, in which expression changes in relevant tissues were registered. (**B**) Tissue-specific and non-specific genes (in any tissue) with the number of terms and categories from the entire pool of HPO terms/categories. (**C**) Haploinsufficient and haplosufficient genes and their TPM sums across all tissues in HPA and GTEx, number of associations with HPO terms and categories and number of GEO samples and tissues, in which expression changes were recorded.

From a total of 4531 HPO genes, 4526 (99.9%) were present in our list of disease genes. The set of phenotypic terms included 8347 unique items, which were assigned to 25 categories corresponding to various body systems or types of manifestation (Supplementary Table S11). For our set of 4526 genes, we compared tissue-specific and non-specific genes (total PBMC, lung, and cerebral cortex) by the number of terms relating to phenotypic categories “Blood and blood-forming tissues”, “Respiratory”, and “Nervous system”, and the number of GEO samples, in which expression changes in the relevant tissues were registered (Figure 5A). A similar analysis was performed for gene sets having associations within phenotypic categories “Musculature” and “Integument” (Supplementary Figure S6). In each series of comparisons, we found highly significant differences. At the same time, when considering the pools of HPO terms/categories and genes enriched/enhanced in any tissue, it turned out that tissue-specific genes had fewer associations compared to tissue non-specific genes (Figure 5B). We next analyzed a subset of haploinsufficient genes (*n* = 841) defined as having haploinsufficiency (HIS) scores ≤ 10% (Supplementary Table S11). HIS genes compared to other disease genes from HPO had (i) higher TPM sums across all tissues in HPA and GTEx, (ii) higher number of associations with HPO terms and categories, and (iii) higher number of GEO samples and tissues, in which expression changes were recorded (Figure 5C). Thus, the level of gene expression in healthy tissues correlated with the number of associations found in DE studies, as well as with the number of phenotypic terms and categories.

## 4. Discussion

In this study, we considered gene expression profiles in healthy tissues and the frequency of gene occurrence as DE in pathology studies and compared these data with a diversity of phenotypic manifestations within a corresponding organ system. We created a set of disease genes using six manually curated gene-phenotype databases. To our knowledge, this is the largest dataset of this kind. With this dataset, we (i) validated the previous findings [6] of higher expression levels of disease genes in pathologically linked tissues than in other tissues, and (ii) demonstrated that disease genes also had higher expression levels than non-disease genes in almost all tissues and blood cells. At the whole genome level, we also showed that (iii) genes with high expression levels in the tissue (in norm) were more likely to be registered in GEO pathology studies in the relevant tissue. Based on the results of GO enrichment analyses for the sets of DE genes in GEO studies, we concluded that (iv) immune response, translation, and tissue-specific processes were the processes most often disturbed in considered tissues and (v) moreover, these down- and up-regulated blocks of genes were frequently found in the same samples, which suggests their compensatory involvement in the same biological processes. Using the subset of disease genes presented in the HPO database, we also established that (vi) the level of gene expression in one/several (for tissue-specific genes) or many (for haploinsufficient genes) healthy tissues correlated with the number of phenotypic terms, i. e. the variety of phenotypic manifestations, within the relevant top-level categories corresponding to organ systems.

Disease genes are often overexpressed in normal tissues where gene defects lead to the development of pathology [6,22]. In confirmation of this phenomenon, we demonstrated higher expression levels for the immune, respiratory, and nervous system disease genes in the corresponding tissues. We also observed that disease genes from our set had higher expression levels than non-disease genes in almost all tissues and blood cells. The disease gene set included highly expressed genes specific for different tissues. Averaging of expression levels in the groups of disease and non-disease genes could partially explain this finding. Beyond that, independently of tissue specificity, genes with relatively high expression levels contribute to the maintenance of tissue regulatory framework and high expression levels may be essential for the functional activity of the tissue [6,23].

In case-control studies comparing transcript levels between healthy and sick individuals, gene expression changes are likely to be a consequence of a disease, rather than its cause or a simple correlation [24]. This hypothesis is consistent with the results of our study, which demonstrated that the disease genes changed their expression not only and not so much in diseases to which they predispose, but also in other pathologies. A relatively small number of gene-phenotype associations supported by expression changes, may be additionally explained by the multifactorial and polygenic nature of the majority of GEO diseases. The disease genes, as well as highly expressed genes in general, were registered as DE genes more often and in more tissues than other genes. Given these results, we supposed that pathology hits highly expressed genes as the main determinants of normal tissue structure and function. Highly expressed genes evolve slowly engaging in multiple interactions in gene-gene networks [25,26,27]. Moreover, highly expressed genes are enriched for biologically important and/or haploinsufficient genes exhibiting higher levels of connectivity compared to other genes [25,28]. High network connectivity was also shown for disease genes [29]. Hence, within tissue interactomes, highly expressed/disease genes might crucially influence dynamic gene expression through their interplay with other genes. The observation of a large number of disease-affected tissues for highly expressed genes is in accordance with the fact that gene expression level correlates with the expression breadth (number of tissues, in which the gene is expressed) [30]. Taken together, these data contribute to understanding of how and why highly expressed/disease genes are frequently dysregulated in pathology studies.

Both down- and up-regulated genes were broadly overrepresented in biological processes relating to immune response, translational control and ribosome biogenesis and tissue-specific perturbations. Our modules did not completely coincide with those identified in the study of Crow et al. [4]. The above-mentioned study was designed to generate a general view from the expression studies, thus all conditions, of which only 25% were determined as diseases, were analyzed together. We did not find overrepresentation of sex and extracellular matrix related genes among preferably DE genes, however, our tissue-based approach allowed us to identify a module that was unique for each tissue. The translational module from our study might be considered interconnected with the modules associated with cell cycle and response to stimulus from the study of Crow et al. [4]. The only consistent result for both studies was the block of DE immune genes.

Due to the direct link between blood and immunity, blood was widely used to study immune-mediated diseases, however, blood is also a useful resource to characterize an individual’s current health status. Peripheral blood contains a wide variety of cells which protect the body from exogenous and endogenous pathogens, thus responding to any alteration of physiological condition of the body and producing messengers between the immune system and other tissues [31]. Multiple diseases, aging, and obesity largely share blood transcriptome profile associated with inflammation and immunity [32]. In our study, immune response genes were also expressed differently in immune and non-immune diseases in tissues other than blood. An expression profile of a damaged tissue reflects tissue infiltration by immune cells implicated in disease development and resolving. Ribosome biogenesis and protein translation was another important block of biological processes often affected in different diseases. These processes are essential for cell growth, proliferation, and response to stress; therefore, their dysregulation affects cellular function thus contributing to cancer and diseases other than cancer [33,34]. As for tissue-specific processes, disease-related expression changes in affected tissues are associated with suppressing and subsequent restoration or compensation of tissue functions. On a more general level, we can speculate that an opposite direction of biological processes may occur as a consequence of the body’s efforts to maintain tissue homeostasis in response to the impact of the stimulus [10,35].

A gene-based comparison of phenotypic diversity in hereditary diseases with the number of expression associations in multifactorial diseases showed their correlation. Phenotypic manifestations of rare diseases are directly related to the gene that is the root cause of the disease, while DE in common diseases reflects the consequences of the disease rather than its prerequisite. However, in any case these results indicate phenotypic impact of the gene at the organ/tissue level. Tissue-specific genes showed organ and tissue-specific effects, while highly and widely expressed haploinsufficient genes exhibited an extended phenotypic impact. The diversity of phenotypic manifestations and DE reflects certain gene properties and can be considered as an indicator of the biological significance of a gene [12].

Among the limitations of the study, it is necessary to note the list of disease genes, which contains most well-characterized genes. Many of them are highly expressed and have broad tissue specificity. GEO datasets may be biased towards better detection of highly expressed genes, as a transcriptome analysis provides more reliable quantitative measurements for highly expressed genes. These limitations reflect a problem which is clearly visible when summarizing the results and is less noticeable in the framework of individual studies aimed at defining DE genes and enriched GO biological processes. Our analysis contributes to the field confirming the need for a nuanced interpretation of DE findings in pathology studies. Our other limitations are related to different methods for the sample and library preparations in GEO studies and the use of the restricted set of GEO samples extracted via a crowdsourcing project. However, this approach also has some strengths, which partially compensate for the shortcomings, since all GEO samples were manually validated for quality [17]. Besides, splitting a series into samples increases the diversity of prioritized genes beneficially influencing the search and interpretation of commonalities and differences in gene–phenotype relationships [36].

## 5. Conclusions

Our results support the conclusion on predictable DE for a specific fraction of genes [4]. These genes are generally highly expressed and are enriched for disease genes. The frequent overlaps between down- and up-regulated BP found in our study are in line with the statement about the predictability of DE of genes belonging to certain functional blocks. These are primarily genes implicated in immune response, translation, and tissue-specific processes. The expression level in healthy tissues correlates with both the number of DE findings and diversity of phenotypic associations in pathology studies. Establishing a link between expression and phenotype contributes to the deeper and more nuanced understanding of the mode of development and progression of human diseases.

## Data Availability

The original contributions presented in the study are included in the article/Supplementary Material.

## Supplementary Materials

The following supporting information can be downloaded at: https://www.mdpi.com/article/10.3390/genes13071168/s1. Supplementary Figure S1: Expression profiles of immune disease related genes, other disease genes and non-disease genes. Genes with TPM < 1 were excluded. FDR corrected *p* values are marked with asterisks: * *p* < 0.05, ** *p* < 0.005, *** *p* < 0.0005, **** *p* < 0.00005. Summary statistics are given in Supplementary Table S3; Supplementary Figure S2: Expression profiles of respiratory disease related genes, other disease genes and non-disease genes. Genes with TPM < 1 were excluded. FDR corrected *p* values are marked with asterisks: * *p* < 0.05, ** *p* < 0.005, *** *p* < 0.0005, **** *p* < 0.00005. Summary statistics are given in Supplementary Table S3; Supplementary Figure S3: Expression profiles of nervous system disease related genes, other disease genes and non-disease genes. Genes with TPM < 1 were excluded. FDR corrected *p* values are marked with asterisks: * *p* < 0.05, ** *p* < 0.005, *** *p* < 0.0005, **** *p* < 0.00005. Summary statistics are given in Supplementary Table S3; Supplementary Figure S4: Box-plots depicting differences in the number of associations in GEO samples for disease genes compared to non-disease genes; Supplementary Figure S5: The results of GO enrichment analysis. (A) Cluster representatives for the most frequent BP dysregulated in skeletal muscle and skin. Cluster representatives for GO terms (terms remaining after reducing redundancy) were obtained using the REVIGO tool. The number of samples for each tissue is indicated in brackets. (B) REVIGO scatterplots for the same BP down-and up-regulated in the same samples. GO terms are plotted according to log size on the *x*-axis and frequency of occurrence (yellow is larger, red is smaller) in the samples on the *y*-axis. The size of the circles is proportional to the underlying frequency of the GO term in a reference database—the EBI GOA database. Functionally similar GO terms are located close to each other; Supplementary Figure S6: Box-plots depicting differences in the number of phenotypic associations within categories Musculature and Integument and gene expression-disease associations in the relevant tissues in GEO for tissue-specific and tissue non-specific genes; Supplementary Table S1: The list of disease genes from OMIM, ORPHANET, DDG2P, IUIS, DisGeNet and MalaCards. The total number of diseases per gene in each database is indicated. The numbers of immune, nervous, and respiratory diseases are specified in accordance with the database classifications. Examples of respiratory, nervous, and ‘other’ diseases include no more than three diseases per gene. An example of a complete list of phenotypes for a given gene is shown for genes associated with immune diseases in Supplementary Table S2. The same disease can belong to different classifications. Columns “Rare-1, Non-rare-2” are present in ORPHANET, DisGeNet and MalaCards blocks. In ORPHANET ‘2’ indicates genes that are associated with only non-rare phenotypes in Europe; in DisGeNet ‘2’ indicates genes that are not associated with OMIM/ORPHANET phenotypes and are not related to Congenital, Hereditary, and Neonatal Diseases and Abnormalities according MeSH classifications; MalaCards ‘2’ indicates genes that are not associated with fetal, genetic or rare phenotypes according to MalaCards global classifications; Supplementary Table S2: The list of immune disease genes with the full list of specific phenotypes identified as related to the immune system in accordance with database classifications; Supplementary Table S3: Summary statistics for a comparison of expression levels of (i) genes involved in the development of immune, respiratory and nervous system diseases, (ii) genes associated with non-immune, non-respiratory and non-nervous system diseases and (iii) non-disease genes. Genes with TPM < 1 were excluded. The highlighted in bold data show that genes involved in the development of immune system diseases had higher levels of RNA expression in all immune related tissues and some other tissues (38/77 in two tissue sets) compared to genes associated with non-immune diseases; Supplementary Table S4: Sensitivity analysis for a comparison of expression levels of (i) genes involved in the development of immune, respiratory and nervous system diseases, (ii) genes associated with non-immune, non-respiratory and non-nervous system diseases and (iii) non-disease genes. Genes with TPM < 1 were excluded. Data are shown for immune, respiratory and nervous system related tissues The highlighted in bold data show results for disease genes in pathologically linked tissues. Adjusted *p* values (*P.adj*) were calculated taking into account all tissues (HPA, *n* = 43; GTEx, *n* = 34) and the number of samples compared. Supplementary Table S5: The list of GEO samples and tissues considered in the study; Supplementary Table S6: The list of differentially expressed (DE) genes in GEO samples with summary statistics data. Enriched genes include genes relating to all categories of enrichment in a corresponding tissue (tissue enriched, group enriched, tissue enhanced); Supplementary Table S7: The list of disease genes linked to the diseases from GEO studies within the Enrichr libraries. The table includes data for genes associated with the same diseases in the gene-phenotype databases considered in the paper and in GEO studies; Supplementary Table S8: The results of GO enrichment analysis with STRING. Dysregulated biological processes were analyzed separately for each sample. The differences in the number of samples between down- and up-regulated genes are due to non-significant enrichment results for some samples; Supplementary Table S9: The results of GO enrichment analysis via STRING processed with REVIGO: cluster representatives for the same BP down-and up-regulated in the same samples; Supplementary Table S10: The results of GO enrichment analysis with Enrichr for the subsets of neoplastic diseases. Disease classification is given in Supplementary Table S5. Dysregulated biological processes were analyzed separately for each sample; Supplementary Table S11: The list of 4526 HPO genes considered in the study. The table includes data on the number of gene—unique phenotypic term associations within 25 categories, haploinsufficiency scores, data on the results from the TissueEnrich analysis and the number of gene expression-disease associations in the Enrichr libraries for GEO disease studies.
