# Supplementary material for: Genes and Diseases: Insights from Transcriptomics Studies
Source: Genes (Basel). 2022 Jun 28;13(7):1168. doi: 10.3390/genes13071168 (PMC9317567; doi:10.3390/genes13071168)
Supplement: Supplementary file 1 [file genes-13-01168-s001.zip › Proofreading_Supplementary Figures.pdf]

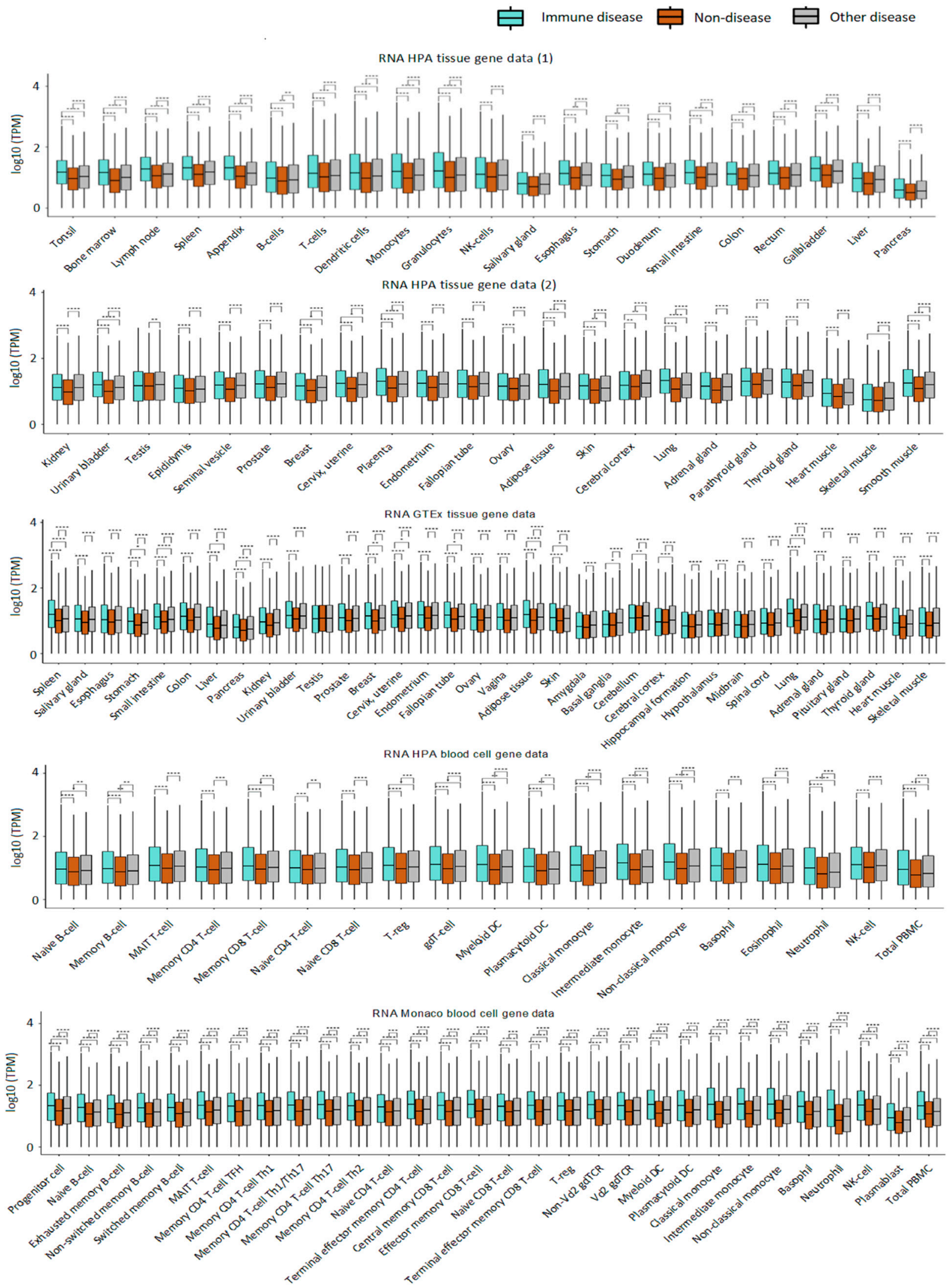

Supplementary Figure S1: Expression profiles of immune disease related genes, other disease genes and non-disease genes. Genes with TPM < 1 were excluded. FDR corrected *P* values are marked with asterisks: $*P < 0.05$, $**P < 0.005$, $***P < 0.0005$, $****P < 0.00005$. Summary statistics are given in Supplementary Table S3.

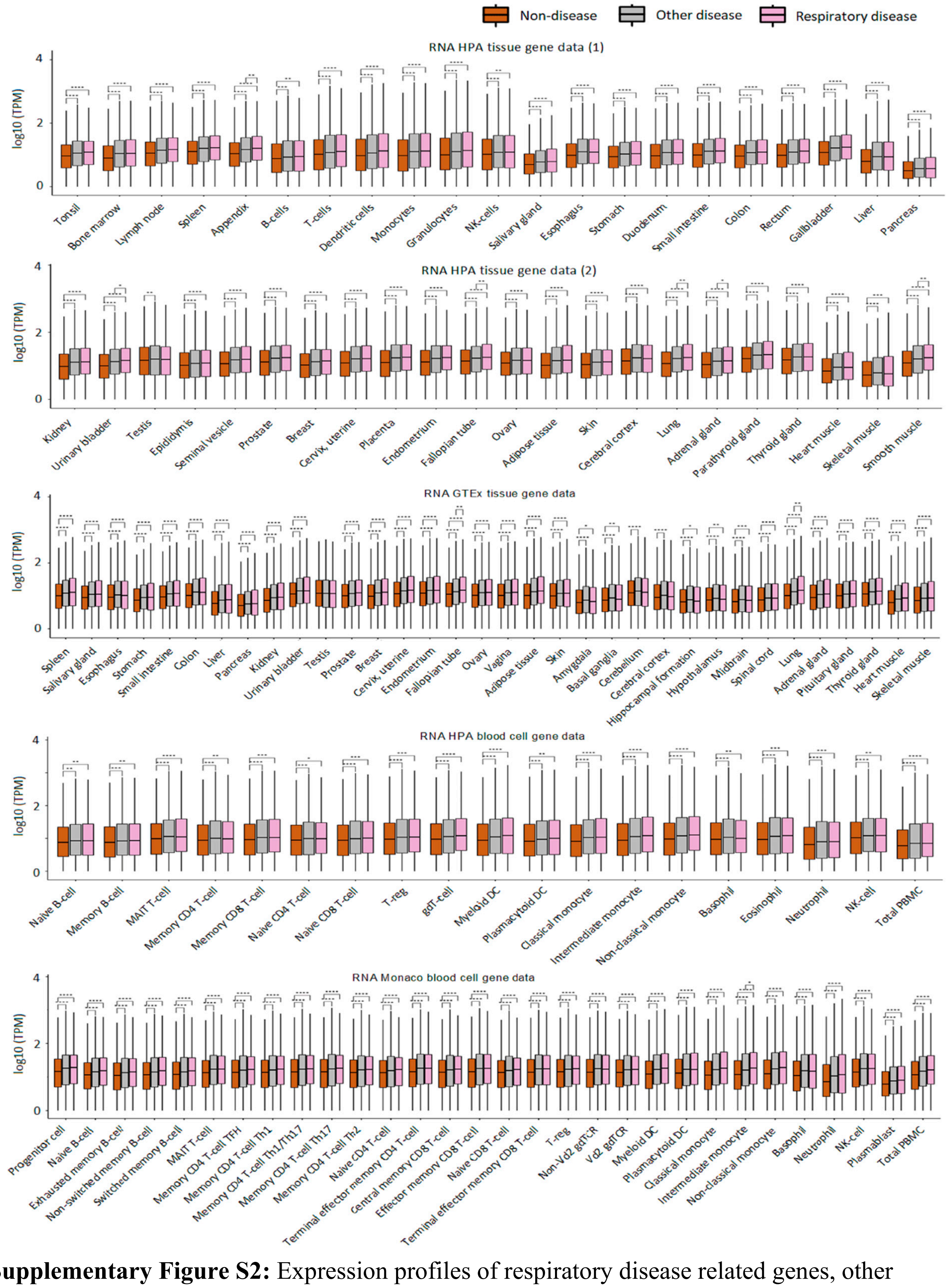

Supplementary Figure S2: Expression profiles of respiratory disease related genes, other disease genes and non-disease genes. Genes with TPM < 1 were excluded. FDR corrected *P* values are marked with asterisks: **P* < 0.05, ***P* < 0.005, ****P* < 0.0005, *****P* < 0.00005. Summary statistics are given in Supplementary Table S3.

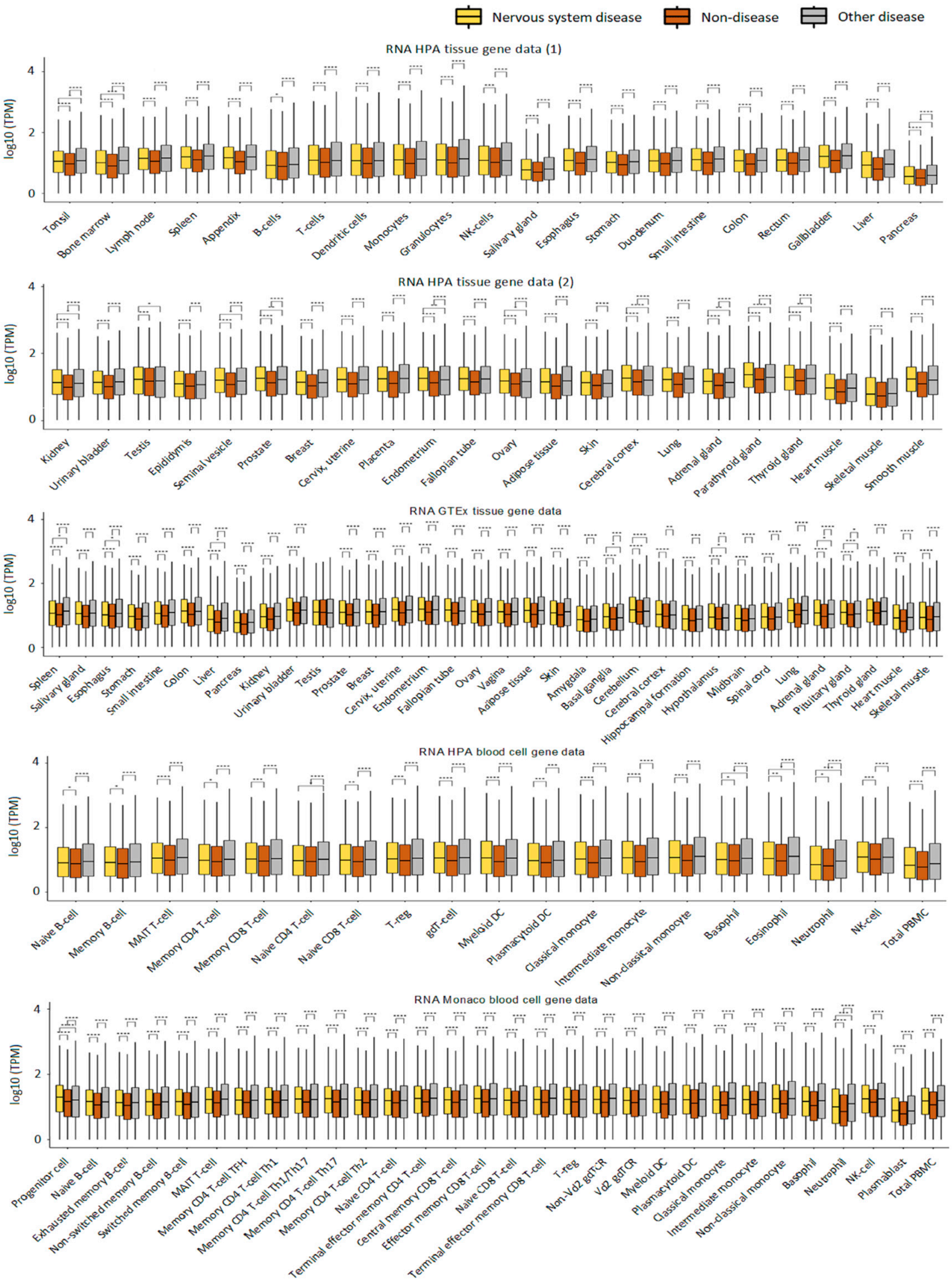

Supplementary Figure S3: Expression profiles of nervous system disease related genes, other disease genes and non-disease genes. Genes with TPM < 1 were excluded. FDR corrected *P* values are marked with asterisks: **P* < 0.05, ***P* < 0.005, ****P* < 0.0005, *****P* < 0.00005. Summary statistics are given in Supplementary Table S3.

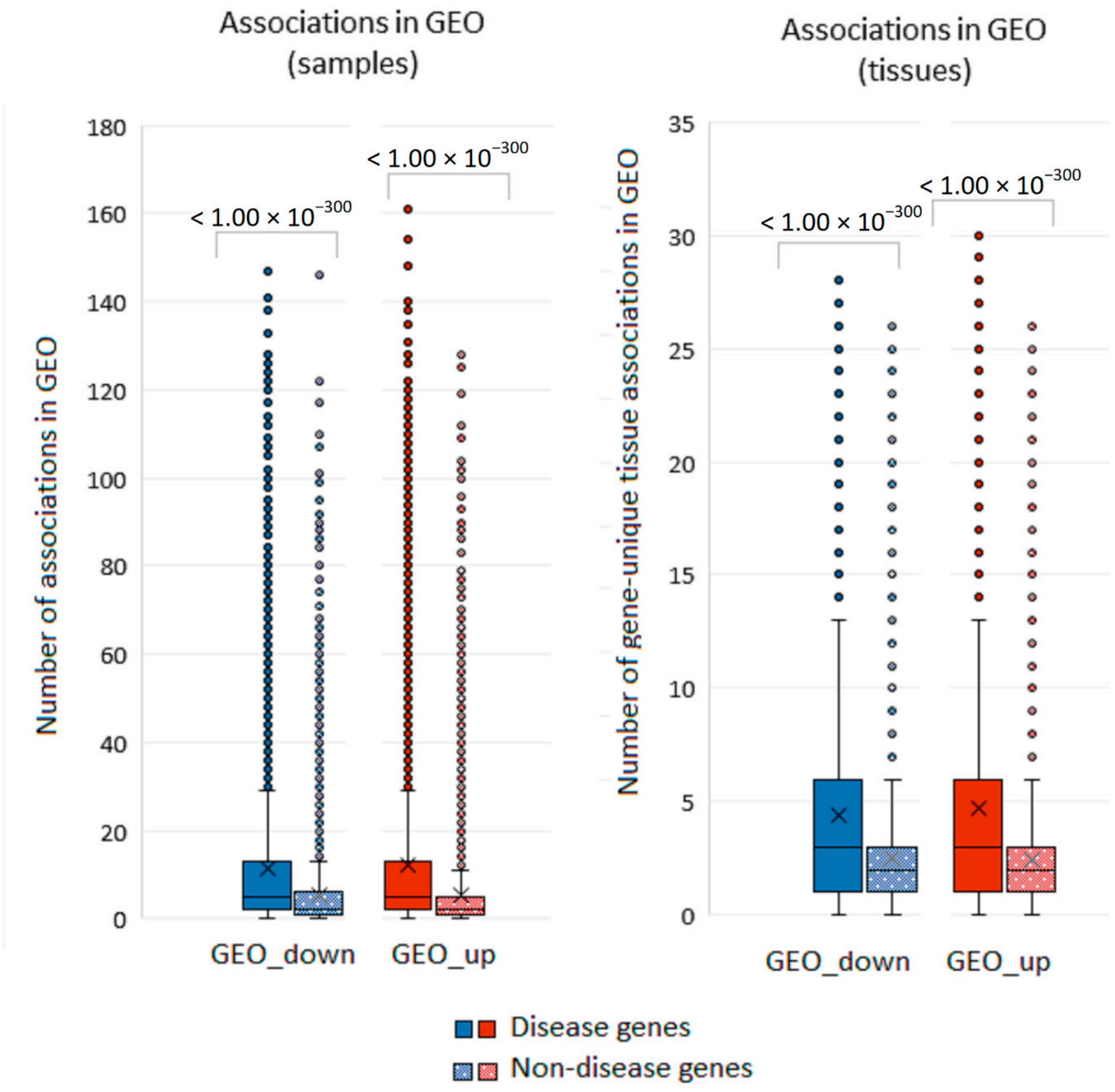

Supplementary Figure S4: Box-plots depicting differences in the number of associations in GEO samples for disease genes compared to non-disease genes.

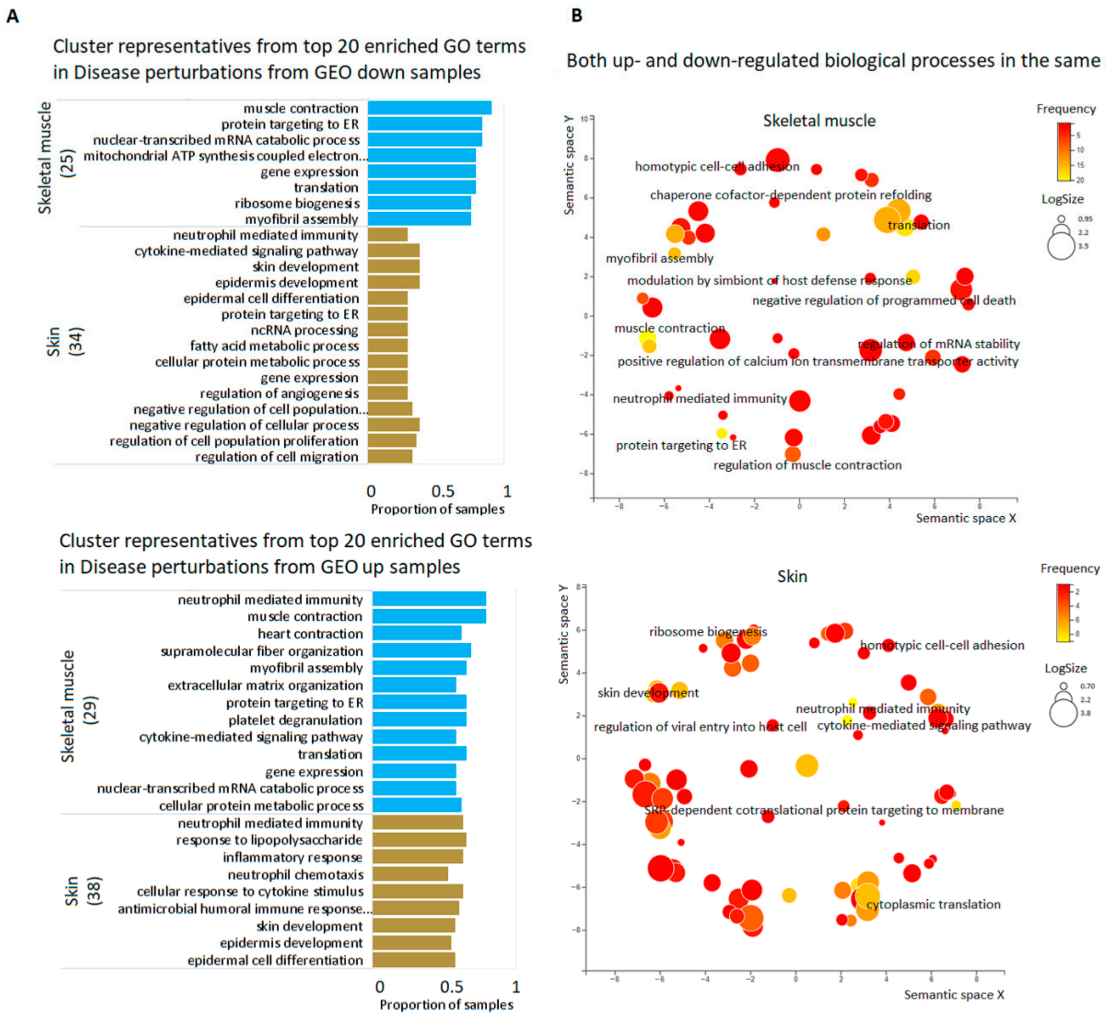

Supplementary Figure S5: The results of GO enrichment analysis. **(A)** Cluster representatives for the most frequent BP dysregulated in skeletal muscle and skin. Cluster representatives for GO terms (terms remaining after reducing redundancy) were obtained using the REVIGO tool. The number of samples for each tissue is indicated in brackets. **(B)** REVIGO scatterplots for the same BP down-and up-regulated in the same samples. GO terms are plotted according to log size on the x-axis and frequency of occurrence (yellow is larger, red is smaller) in the samples on the y-axis. The size of the circles is proportional to the underlying frequency of the GO term in a reference database – the EBI GOA database. Functionally similar GO terms are located close to each other.

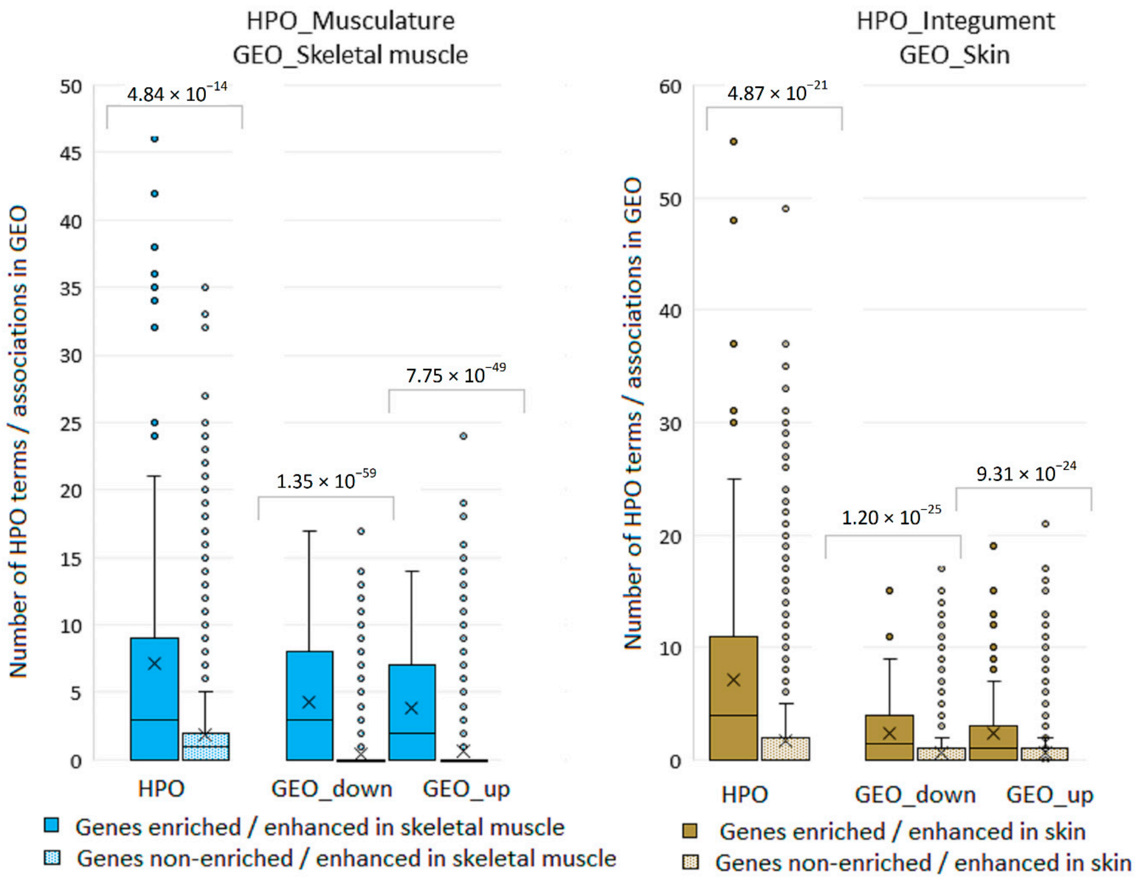

Supplementary Figure S6: Box-plots depicting differences in the number of phenotypic associations within categories Musculature and Integument and gene expression - disease associations in the relevant tissues in GEO for tissue-specific and tissue non-specific genes.
